# Errata

**Published:** 1884

**Authors:** 


### ERRATA.

Page 15, 1st column, line 13 from bottom, for Arn.$^{7}$ read Amm. c.$^{7}$

Page 29, 2d col., line 19 from bottom, omit Euph.$^{7}$

Page 32, 2d col., line 7 from bottom, for Min. ac. read Mur. ac.; line 8, for Mag. read Mez.

Page 33, 1st col., line 12 from bottom, for Eupin read Eupion.

Page 34, 1st col., line 19 from top, for expectoration read expiration; *same* at page 43, 2d col., line 9th.

Page 36, 2d col., line 21, for Aug. read Ang.

Page 37, 2d col., line 17 from bottom, for Dig. read Dign.

Page 40, 1st col., line 14 from bottom, for Sepia read, (6-7 P. M.) Ipec.

Page 42, 1st col., line 25, for Phal. read Phell.

Page 43, 2d col., line 4 from bottom, for EUPH. read EUPHR.

Page 48, 1st col., line 4, for Aqu. fr. read Aqu. petr.

Page 48, 2d col., line 4 from bottom, for Hell. ox. read Hell. or.

Page 49, 2d col., line 19, for Sil. t. read Lil. t.

Page 51, 1st col., line 10th from bottom, for Hydras. read Hyper.$^{7}$; *same* at p. 115, line 20, 2d col.

Page 52, 1st col., line 17 from bottom, for Ranc read (Raue).

Page 56, 2d col., line 3 from bottom, for Stam. read Stram.

Page 61, 2d col., line 21, for *Cori v.* read *Coral.*

Page 62, 1st col., line 14, for Merc. ac. read Mur. ac.

Page 63, 1st col., line 30, for agg. read amel.

Page 68, 1st col., line 3, for day read dry.

Page 68, 1st col., line 13, for Coc. c. read Cocc.$^{6}$

Page 68, 2d col., line 17, for Coloc. n., read Colocn.

Page 80, 2d col., bottom line, for Aur. mur.$^{7}$ read Am. mur.$^{7}$

Page 90, 1st col., line 3, for amel. read aggr.

Page 91, 2d col., line 9, for sir read air.

Page 98, 1st col., line 3d from bottom, omit Kreos.

Page 102, 2d col., line 3 from bottom, for Lach. v.$^{2}$ read Lact. v.$^{2}$

Page 106, 1st col., 2d line, for *when* read *causes.*

Page 109, 1st col., line 11 from bottom, for D5.ig. read Dig.—5.

Page 119, 1st col., line 22, for Mag. s.$^{1}$ read Mag. c.$^{1}$

Page 131, 2d col., line 15, for Dulch. read Dulc.

Page 150, 1st col., line 5, for An.$^{7}$ read Arn.$^{7}$

